# Creating insect neopolyploid lines to study animal polyploid evolution

**DOI:** 10.1111/eva.13706

**Published:** 2024-09-08

**Authors:** Saminathan Sivaprakasham Murugesan, Leo W. Beukeboom, Eveline C. Verhulst, Kelley Leung

**Affiliations:** ^1^ Laboratory of Entomology Wageningen University & Research Wageningen The Netherlands; ^2^ Groningen Institute for Evolutionary Life Sciences University of Groningen Groningen The Netherlands

**Keywords:** development and evolution, evolution of sex, evolutionary theory, experimental evolution, life history evolution

## Abstract

Whole‐genome duplication (polyploidy) poses many complications but is an important driver for eukaryotic evolution. To experimentally study how many challenges from the cellular (including gene expression) to the life history levels are overcome in polyploid evolution, a system in which polyploidy can be reliably induced and sustained over generations is crucial. Until now, this has not been possible with animals, as polyploidy notoriously causes first‐generation lethality. The parasitoid wasp *Nasonia vitripennis* emerges as a stunningly well‐suited model. Polyploidy can be induced in this haplodiploid system through (1) silencing genes in the sex determination cascade and (2) by colchicine injection to induce meiotic segregation failure. *Nasonia* polyploids produce many generations in a short time, making them a powerful tool for experimental evolution studies. The strong variation observed in *Nasonia* polyploid phenotypes aids the identification of polyploid mechanisms that are the difference between evolutionary dead ends and successes. Polyploid evolution research benefits from decades of *Nasonia* research that produced extensive reference—omics data sets, facilitating the advanced studies of polyploid effects on the genome and transcriptome. It is also possible to create both inbred lines (to control for genetic background effects) and outbred lines (to conduct polyploid selection regimes). The option of interspecific crossing further allows to directly contrast autopolyploidy (intraspecific polyploidy) to allopolyploidy (hybrid polyploidy). *Nasonia* can also be used to investigate the nascent field of using polyploidy in biological control to improve field performance and lower ecological risk. In short, *Nasonia* polyploids are an exceptional tool for researching various biological paradigms.

## 1 INTRODUCTION

Polyploidy is the multiplication of chromosome sets through whole‐genome duplication (WGD). Autopolyploidization is intraspecific WGD, and allopolyploidization is the hybridization and genome combination of different parental species (Spoelhof et al., 2017). Ancient polyploids are taxa with an ancestral polyploidization event followed by genome reduction to re‐establish diploidy, widely represented among plants and animals (Mable, 2004). Polyploidy can spur evolution of complex gene networks and mass speciation and adaptive advantage to abiotic stress (Comai, 2005; Wertheim et al., 2013). It is thus a major evolutionary driver, but paradoxically, it is also highly deleterious to the individual. Neo‐polyploids often suffer deleterious sterility, gene expression disruption, and gigantism from the cellular to organismal level (Comai, 2005). How short‐term polyploid disadvantages are overcome to confer long‐term advantage is a longstanding question in evolutionary biology. For animals, polyploidy's eco‐evolutionary consequences have been most prevalently studied in fish, amphibians, and reptiles (Mable, 2004; Otto & Whitton, 2000; Soltis et al., 2016). However, due to the difficulty of inducing polyploidy and maintaining it over generations, many studies have focused on genomic signatures of ancestral polyploidy or single‐generation neopolyploid effects. The inability to conduct multigenerational experimental evolution studies has limited our understanding of how animal neopolyploids bypass initial disadvantages (Baduel et al., 2018; Mable, 2004; Spoelhof et al., 2017).

Because the incidence of ancestral WGD varies among animal groups, so does the evolutionary contribution of polyploidy. In insects, polyploidization was thought to occur in only a few species capable of self‐reproduction, as their doubled‐chromosome gametes would no longer form zygotes with gametes of normal individuals (Engel, 2015; Li et al., 2018; Lokki & Saura, 1979). However, a phylogenomic study revealed that many insects had undergone WGD and that many of the same genes are retained across independent WGD/subsequent re‐diploidization events (Li et al., 2018). This illustrates the power of using insect systems to make broader inferences on animal polyploid evolution, akin to how *Arabidopsis* (Fernandes Gyorfy et al., 2021) and duckweed (Glennon et al., 2014) polyploids were used to more broadly understand plant evolution.

One insect order with special utility in animal polyploid research is the Hymenoptera (bees, wasps, ants, and sawflies). Because of their haplodiploid sex determination, disparate ploidy levels are inherent. Males develop from unfertilized eggs that are haploid, and females develop from fertilized eggs that are diploid. Hymenopteran neopolyploids (diploid males, triploid females) are fairly prevalent across distantly related species (Leung et al., 2019; van Wilgenburg et al., 2006). However, most still have first‐/second‐generation polyploid sterility and so still cannot be used for experimental evolution (Leung & van der Meulen, 2022). Furthermore, there are problems of adequate sample size, as polyploids are typically produced at low numbers. A striking exception is *Nasonia vitripennis* Walker (Hymenoptera: Pteromalidae), a parasitoid wasp that reproduces on blowfly pupae. Its ease of use as a laboratory model has spawned a large body of work on behavior, genetics, development, evolution, and ecology (Lynch, 2015; Mair & Ruther, 2019; Wang et al., 2013; Werren et al., 2010). Notably, *Nasonia* polyploidy has been known and used for research since the 1940s (Beukeboom & Kamping, 2006; Geuverink et al., 2017; Koevoets et al., 2012; Leung et al., 2023; Verhulst, 2010; Whiting, 1960; Zou et al., 2020). Here, we present *Nasonia*'s potential to study polyploidy‐linked biological paradigms, starting with methods of creating reproductively competent neopolyploids for longer‐term evolutionary studies S1.

## 2 *NASONIA*: A VERSATILE HYMENOPTERAN MODEL

The convenience of the genus *Nasonia* as a model (Werren & Loehlin, 2009) is a major aspect of its benefit to polyploid research. The four species *N. vitripennis, N. giraulti, N. longicornis*, and *N. oneida* can all be reared on commercial blowfly hosts (Raychoudhury et al., 2010; Werren et al., 2010). Cosmopolitan *N. vitripennis* is the flagship species for research. The four species can be intercrossed if they are cured of their *Wolbachia* endosymbiotic bacteria, a prezygotic species barrier (Werren et al., 2010). A culture cycle of ~14 days at 25°C facilitates breeding a large number of generations within a short time frame (Werren & Loehlin, 2009), increasing the likelihood of observing significant evolutionary effects in a relatively short experimental period. Large sample numbers can be obtained as single females typically produce 30–40 offspring per host (~90% female offspring if mated, only male offspring if unmated) (Werren & Loehlin, 2009). During the pupal stage, individuals can be sexed and sorted as virgins for controlled crosses. Active study and culture workload can be paused as *Nasonia* can be refrigerated at 4°C as pupae to delay development for weeks. Moreover, female wasps exposed to winter‐like conditions of short photoperiod and cold temperature (8L:16D, 18°C) produce diapause larvae in developmental halt, which may be kept in cold storage for up to 2 years. Normal development resumes with the reintroduction of longer light and higher temperatures (16L:8D, 25°C). Advanced genetic and genomic tools and resources have been developed for *Nasonia*, including high‐quality genomes and RNA interference to study gene function (Lynch & Desplan, 2006; Werren et al., 2010; Werren & Loehlin, 2009). Relatively small genomes (~300 MB, five chromosomes) (Beukeboom & Desplan, 2003) make transcriptomics and re‐sequencing objectives cheaper and faster than other animal systems. Haploid males allow for easy screening of recessive mutations, and phenotypic markers on all five chromosomes assist Mendelian cross experiments (Beukeboom & Desplan, 2003; Loehlin et al., 2010; Lynch, 2015).

### 2.1 A long‐established *Nasonia* polyploid line

**FIGURE 1:**
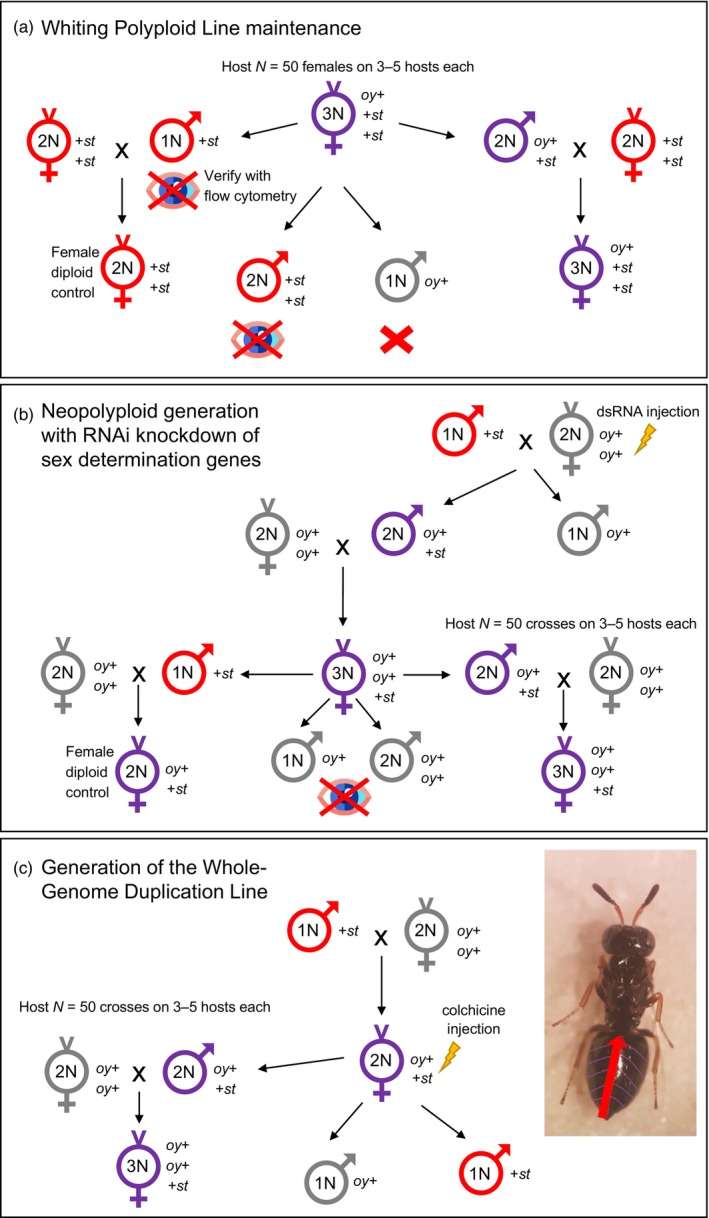
Polyploid line generation and maintenance. For (a) Whiting polyploid line (WPL) maintenance, WPL 3N virgin females produce four types of male offspring: red‐eyed haploid males, red‐eyed diploid males, oyster‐eyed (gray) haploid males, and purple‐eyed diploid males. To produce purple‐eyed triploid females, the purple‐eyed diploid males are separated using an eye color marker and then mated with STDR strain females, which has red‐eye markers. In red‐eyed males, diploids comprise ~25% of individuals and cannot be visually distinguished from haploids; flow cytometry is needed. For diploid female controls to compare to triploid females, ~75% of red‐eyed haploid males can be mated to an STDR female, but post hoc flow cytometry should be used to confirm ploidy. Oyster‐eyed males no longer occur due to the marker's weak phenotypic penetrance. For (b) neopolyploid generation with RNAi knockdown of sex determination genes, inject oyster strain 2N virgin female white pupae mounted with glue on a glass slide with dsRNA for target sex determination gene. Place on 5% agar and in normal culture conditions to allow continued development. Mate with males of the STDR line. The recommended number of wasps to inject for getting at least *N* = 150 diploid male‐producing females are *transformer N* = 300, *transformer 2N* = 600, *wasp overruler of masculinization N* = 400. Oyster‐eyed male offspring are haploid. Purple‐eyed are the diploidized males and are mated to oyster females to produce purple‐eyed neotriploids. These virgin purple‐eyed females produce red‐eyed haploids males that can be crossed to oyster females to create diploid female controls for the triploid females, oyster‐eyed haploids and diploids that are visually indistinguishable that may be discarded from the study, and diploid purple‐eyed males. For (c) generation of whole‐genome duplication Line (WGDL), inject 1‐day‐old adult F1 oyster/STDR hybrid females with a heat‐dissolved solution of 0.5 M colchicine using a Femtojet 1 microneedle. Injection requires narcotization of the female on a CO2 pad, and pinning the female dorsal side down by the wings with microtweezers in one hand. With the other hand, slide a microneedle under the first or second abdominal plate junction (red arrow on the wasp photograph) and inject using a foot pedal attachment (without this attachment, two people are needed, one to hold down the female and handle the needle, and the other to manually push the injection button on the machine).

Polyploidy in *Nasonia* was first observed in the 1940s when it spontaneously appeared in laboratory stocks. A long‐maintained inbred line, the Whiting polyploid line (WPL), was derived from this unknown mutation (Leung et al., 2019, 2023; Whiting, 1960). This line may be considered representative of evolutionary adaptation to polyploidy over many generations. WPL polyploids produce viable and fertile offspring. Diploid males produce diploid sperm mitotically and have high fecundity, whereas triploid females produce a few offspring by meiotic oogenesis, sufficient for continuing the line (Leung et al., 2019, 2023; Whiting, 1960). With scaling up of the affordable and easy *Nasonia* culture process, many polyploids can be produced for larger‐scale studies. Two lines with eye mutations that are in complementation were incorporated to quickly distinguish WPL polyploids and nonpolyploids, eliminating the need for flow cytometry or karyotyping. The polyploid culture cycle alternates between diploid males and triploid females. The male generation of the WPL culture scheme has gray‐eyed (also called oyster) haploid males, red‐eyed males that are a mix of haploid and diploid (but are predominantly haploid) and purple‐eyed (wildtype color) diploid males. These purple‐eyed diploid males are mated to red‐eyed diploid females of the STDR red‐eye mutant line to produce purple‐eyed triploid females. The triploid females then produce ~25% diploid, ~75% haploid males when hosted as virgins (diploid female counterparts can be made by outcrossing WPL haploids to diploid females from a stock line separately maintained from WPL) (Beukeboom & Kamping, 2006; Leung et al., 2019; Whiting, 1960). Full WPL culture details, including eye marker genotypes and corresponding phenotypes, are in Figure 1a.

### 2.2 Polyploids made with single‐gene knockdowns of the sex determination cascade

More recently, it became possible to create *Nasonia* neopolyploids that can be maintained over multiple generations by using RNA interference to knockdown different genes of *Nasonia's* female sex determination pathway: *transformer* (*tra*) (Verhulst, 2010), *transformer‐2* (*tra2*) (Geuverink et al., 2017), and *wasp‐overruler‐of‐masculinization* (*wom*) (Zou et al., 2020). Polyploids are obtained by microinjecting double‐stranded RNA into female pupae, allowing them to mate with males as adults, and screening for diploid male offspring that have been diverted from female development.

Besides the masculinization of diploid zygotes, each gene knockdown has distinct effects. *Tra* knockdown produces a high number of diploid males but also a large number of diapausing larvae that cannot be sexed (Leung et al., 2023; Verhulst, 2010). Knockdown of *tra2* in *N. vitripennis* causes high juvenile mortality, indicative of an essential role in early embryonic development (Geuverink et al., 2017). *Wom* is a regulatory gene upstream of *tra* in the sex determination cascade of *N. vitripennis*, and it is responsible for initiating zygotic *tra* expression (Verhulst et al., 2013; Zou et al., 2020). Knockdown of *wom* does not result in high larval diapause like *tra* knockdown (Verhulst, 2010) or high juvenile mortality like *tra2* knockdown (Geuverink et al., 2017). This variation in knockdown effects suggests each sex determination gene influences different downstream gene networks, likely translating to variation in polyploid evolution outcomes.

For *N. vitripennis*, we recommend making neopolyploids using the same mutant eye markers as WPL. The caveat is that WPL was created with the red‐eye mutant line (STDR) for the female generation, but we observed a low female mating rate in this line following lab adaptation. To increase line establishment likelihood and sample size of subsequent generations, the oyster (gray) eye mutant line should be used instead. The full knockdown line creation and maintenance protocol are in Figure 1b. In short, these lines have oyster (gray) eye males that may be either haploid or diploid, and so are not used in assays or crosses, so we use the red‐eyed haploid males and purple‐eyed diploid males. The female generation has purple‐eyed triploid females, like WPL.

### 2.3 Polyploids made by whole‐genome duplication using colchicine

Polyploidization through single‐gene knockdown of *Nasonia* sex determination genes differs from polyploidization methods used in other systems. Those polyploidization modes rely on meiotic failure, in which duplicated chromosomes do not segregate, producing unreduced gametes. They presumably represent ancestral polyploid events. We, therefore, produced an analogous *N. vitripennis* whole‐genome duplication line (WGDL).

For other animals, polyploidization through meiotic failure has been achieved through temperature stress, mechanical stress, and chemical stress. We attempted to induce polyploidy in *Nasonia* using heat shock (Kawamura, 1994) and CO_2_ narcosis (Oldroyd et al., 2018) protocols from other insect systems but were not successful. Instead, we created a WGD event through chemical induction. Chemical polyploidization through meiotic failure is well established in plant research, such as with colchicine (Dermen, 2014), preventing spindle formation. Until now, no animal polyploids with reproductive capacity have been created with colchicine. Through colchicine injection of adult female wasps hosted as virgins, we created a single diploid male offspring through meiotic failure. It was used to establish the *N. vitripennis* WGDL. Like the single‐gene knockdowns, the WGDL uses a cross‐scheme with red‐eyed haploid males, purple‐eyed diploid males, gray‐eyed males discarded for ploidy uncertainty, and purple‐eyed triploid females. Notably, colchicine WGD induction is infrequent, but a single event can result in many offspring. The full colchicine WGDL generation and cross‐maintenance protocol is in Figure 1c.

## 3 ANTICIPATED RESEARCH DIRECTIONS AND APPLICATIONS OF *NASONIA* POLYPLOIDS

### 3.1 Gene expression changes

**FIGURE 2:**
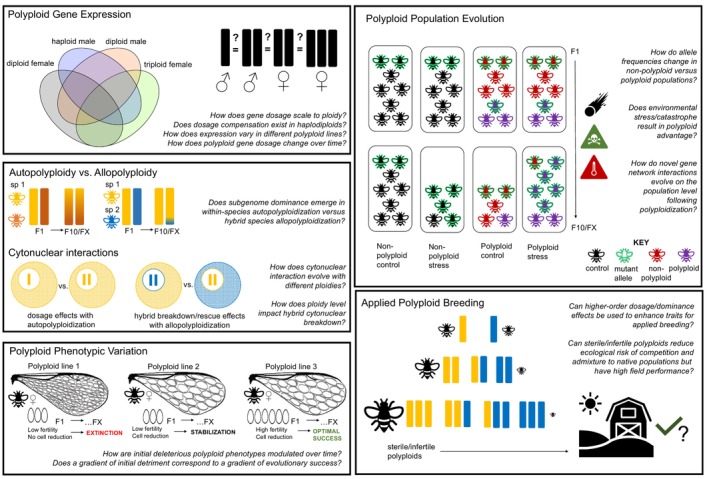
Research possibilities with *Nasonia* polyploids. Each bar represents a whole‐genome set. Text in italics are possible research questions but are not exhaustive.

Here we describe extensive research avenues made possible with *Nasonia* neopolyploids (summarized in Figure 2). These possibilities are not exhaustive, and we expect further lines of inquiry to follow from our suggestions. A consistent theme is how additional genome sets change gene expression (Comai, 2005). Particularly, disruption of sexual dosage mechanisms that balance the expression of sex chromosomes between males and females seems to be why obligate sexual species survive polyploidization less than those capable of reproducing asexually or with selfing (David, 2022). The mechanism by which the sexual species (e.g., the two WGDs at the base of vertebrate evolution; Dehal & Boore, 2005) survived their ancestral polyploidization has been unaccounted for. *Nasonia* lacks sex chromosomes, although interestingly, there is some evidence for absolute expression being conserved across sex rather than ploidy (Leung et al., 2023)—this in itself is ground for investigating the major evolutionary question of whether haplodiploids have sexual dosage compensation across all chromosomes. Thus, *Nasonia* neopolyploids present a rare opportunity to study polyploid gene expression in sexual species postpolyploidization without lethal or sterilizing sexual dosage compensation effects (Leung et al., 2023). As the *Nasonia* genome is small, it is also easier to study how genomes reduce in size after the genome rediploidizes (e.g., which elements are eliminated first and why) via re‐sequencing of generations following neopolyploidization.

### 3.2 Autopolyploidy versus allopolyploidy

Both auto‐(intraspecific) and allo‐(interspecific) polyploidization events are possible with the *Nasonia* system. So far, we have focused on autopolyploidization of *N. vitripennis*, the flagship research species. However, both polyploidization forms are subject to subgenome evolution research, on which genome becomes “dominant” in genomic content or expression level (Schiavinato et al., 2021). The availability of distinct *N. vitripennis* lines benefits study design for subgenome research as lines can be easily sequenced. Our protocol for creating lines with eye markers to track ploidy already involves two commonly used *N. vitripennis* mutant lines. However, the ease of producing hybrids also makes *Nasonia* a system by which allopolyploids can be made, which have already been used to study hybrid breakdown (Koevoets et al., 2012; Niehuis et al., 2008). This means that the *Nasonia* neopolyploid system can be the first (to the authors' knowledge) to directly compare subgenome evolution within species (autopolyploidy) to that of hybrid genomes (allopolyploidy). It might be particularly interesting to study how each subgenome evolves in interaction with the mitochondrial genome, which can only represent the maternal background (Schiavinato et al., 2021).

### 3.3 Cytonuclear interactions

*Nasonia* polyploids are also a powerful tool for studying cytonuclear effects. This is a fundamental tenet of cellular and developmental biology concerning how the nuclear genome interacts with the separate mitochondrial genome (Koevoets et al., 2012; Telschow et al., 2019). It is unclear how the relative expression dosage of these two genomes is regulated to support the switchover from dependency on maternal transcripts in the oocyte cytoplasm (maternal expression) to expression of the individual's genome itself (zygotic expression). There is much to be learned from the dysregulation of these processes through *Nasonia* polyploids. Neopolyploids have already been used to determine that ploidy level does not play an essential role in the maternal‐to‐zygotic expression transition (Arsala & Lynch, 2017). However, there is evidence in plants (*Arabidopsis*) that organelle genome copy number increases with neopolyploidization, suggesting that a stochiometric balance effect is implemented immediately (Fernandes Gyorfy et al., 2021). *Nasonia* neopolyploids can be used to evaluate this mechanism in depth in animals, particularly given the existing reference knowledge on its mitochondrial genome (Telschow et al., 2019). Finally, cytonuclear interactions are known to factor in species barriers, driving F2 generation breakdown in *Nasonia* hybrids (Koevoets et al., 2012; Telschow et al., 2019). *Nasonia* polyploids add an interesting dimension to what can happen when an additional nuclear genome of one of the parental species is added. Does this aggravate or rescue cytonuclear incompatibilities? Is polyploidization thus a means through which hybridization can be stabilized?

### 3.4 Variation in polyploid phenotypes

*Nasonia* does not have the stereotypical sterility of animal polyploids, but interestingly, the different polyploid lines are also highly variable in fitness (Leung et al., 2023). They produce different numbers of viable offspring and vary in their mating success. The impacts of polyploidy can be studied for potential impacts on pheromones, courtship behaviors, male competition, female mate choice, and sperm usage choice, all of which have already been characterized in *Nasonia* (Benetta et al., 2021; Mair & Ruther, 2019). Another problem of polyploidy is larger cells. This interferes with surface area to volume ratio in function from the cellular to organ levels, and whole‐body gigantism brings challenges of gravity and body plan constraints (Comai, 2005). In other animals, cell number reduction is a mechanism for maintaining normal size and physiology, but this is not well understood in invertebrates (Comai, 2005). *Nasonia* polyploid lines exhibit different phenotypes for cellular size and number (Leung et al., 2023), which enables investigation of when cell reduction occurs, how, and why some organisms and not others use it. It is also completely unknown at this point how whole organism polyploidization impacts inherently endopolyploid cells such as thoracic flight muscle cells in haploid males (Aron et al., 2005), and nurse cells of diploid female ovarioles (Lynch et al., 2011). Determining how these various mechanisms differ among the lines and tracking their long‐term evolutionary effect may be instrumental in elucidating which mechanisms are essential for animal polyploid success.

### 3.5 Polyploid population evolution

One area of polyploid research not possible for *Nasonia* is natural population dynamics. Other studies in polyploid plants and animals have, for example, examined population‐level competition with nonpolyploids or a correlation to environmental conditions (Glennon et al., 2014; Novikova et al., 2020). There is no evidence that *Nasonia* polyploids occur in nature, so these topics cannot be explored. However, artificial polyploid populations are a powerful tool. The inbred lines described up to this point lack genetic variation, but the outbred *N. vitripennis* population HVRx exists (van de Zande et al., 2014). We introgressed eye mutations into this background to allow for the same crossing schemes described in Figure 1 and maintain the genetic variation that can be selected upon in evolutionary processes. Compared to nonpolyploid control populations, such populations can be used to test hypotheses such as the rate of evolution of polyploid populations with accelerated gene network diversification and speciation (Wertheim et al., 2013), stress adaptation to temperature extremes or toxins (Glennon et al., 2014; Novikova et al., 2020), and allele frequency/haplotype changes (De Silva et al., 2005). This can be done efficiently with transcriptomics and whole‐genome sequencing of early and later *Nasonia* polyploid populations. An important caveat is that only the diploidized genome of the polyploid male is kept intact. Outcrossing with a diploid female contributes a nonpolyploidized chromosome set necessary to continue the line with triploid offspring. However, this opens up an interesting means to test a major outstanding question on haplodiploid population genetics. It is presumed that deleterious alleles are quickly purged in the haploid state, making haplodiploid species more resilient to inbreeding depression. However, it has been proposed that many lost alleles are only deleterious to haploid males, disadvantaging diploids if these alleles were beneficial to females (Leung et al., 2020; Miller & Sheehan, 2023). An artificial polyploidized *Nasonia* population could be used to investigate if heterozygous males are disproportionately common over homozygous diploid males, and females exhibit a phenotypic outcome of beneficial alleles retained through the heterozygote male. This would make a strong case for a sexual tradeoff in haplodiploid inbreeding.

### 3.6 Applied breeding

*Nasonia* neopolyploids also present a novel research direction of using polyploidy for applied breeding. Plant polyploidization has long been used to increase crop size and hardiness (Udall & Wendel, 2006). It is also used in some animal aquaculture to produce larger individuals for consumption or aesthetics (Zhou & Gui, 2017). *Nasonia* can be used to pioneer the use of polyploidy in the lucrative industry of biocontrol, the practice of using whole organisms to control pests in agriculture. Biological control is dominated by parasitoid use. As a genus that targets over 68 host species (Whiting, 1967), *Nasonia* is a developed model for biocontrol research. It has already been proposed that *Nasonia* parasitoids be used to test the possibility of beneficial dosage/dominance effects (Leung et al., 2019). If a female can carry three copies of an allele linked to better biocontrol performance rather than two, is that trait further enhanced, for example? If a line has an advantage in the diploid female state that is hemizygous lethal in the typical haploid male, can this trait be rescued by maintaining it in heterozygous diploid males? For hybrids, are there possibilities of using higher ploidy levels to optimize the best traits from both parental species? Last, there is mounting concern about the environmental risk represented in commercial insect populations admixing with natural populations. Mass‐producing sterile insects that can nevertheless perform their field function are common for other species. Although not completely sterile, the many polyploidization modes for *Nasonia* and the large production of polyploid females (the pest‐killing sex) puts it in a strong position as a model for exploring polyploid utility in biocontrol.

## 4 SYNTHESIS

Polyploidy corresponds to the complexification of gene networks, speciation, and stress survival in eukaryotic evolution (Comai, 2005; David, 2022; Soltis et al., 2016). It is highly understudied relative to its importance. Part of this is due to the inherent difficulty of inducing polyploidization and being able to study effects afterward, as it greatly impairs the individual (Mable, 2004; Wertheim et al., 2013). Here we present the parasitoid *Nasonia* as the critically needed system for which polyploidy can be induced and sustained over generations. It emerges as a well‐suited model to comprehensively study the evolutionary paradigms of polyploidy across cellular, gene expression, life history, population genetics, and applied levels.

## CONFLICT OF INTEREST STATEMENT

We have no conflict of interest to declare.

## FUNDING INFORMATION

The writing of this manuscript was funded by the Dutch Research Council (NWO), OCENW.KLEIN.333, which did not influence its contents.

## Supporting information


Figure S1

## Data Availability

It is not applicable, as this is a perspectives paper with no original data.
