# Supplementary figures and images for: Creating insect neopolyploid lines to study animal polyploid evolution
Source: Evol Appl. 2024 Sep 8;17(9):e13706. doi: 10.1111/eva.13706 (PMC11381576; doi:10.1111/eva.13706)

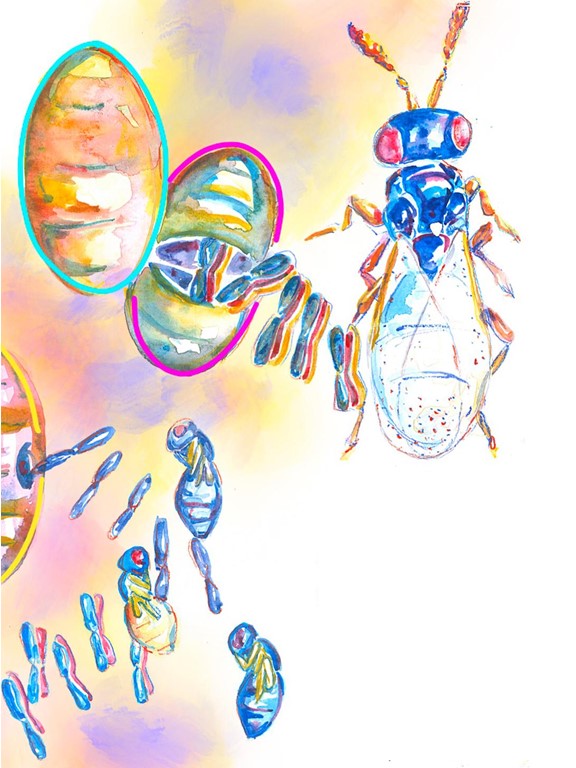

Supplement: Supplementary file 1 — Figure S1 [file EVA-17-e13706-s001.jpg]
